# Frequency of Positive Polymerase Chain Reaction (PCR) Testing for *Borrelia burgdorferi* on Whole Blood Samples That Tested Positive for *Babesia microti* by PCR from an Endemic Area for Both Infections in New York State

**DOI:** 10.3390/pathogens12081066

**Published:** 2023-08-21

**Authors:** Guiqing Wang, Jian Zhuge, Gary P. Wormser

**Affiliations:** 1 Department of Pathology, New York Medical College, Valhalla, NY 10595, USA; hank.wang@nyulangone.org; 2 Department of Pathology and Clinical Laboratories, Westchester Medical Center, Valhalla, NY 10595, USA; jian.zhuge@wmchealth.org; 3 Division of Infectious Diseases, New York Medical College, Valhalla, NY 10595, USA

**Keywords:** Lyme disease, *Babesia microti*, tick-borne diseases, babesiosis, coinfection, polymerase chain reaction, *Borrelia burgdorferi*

## Abstract

Because both *Babesia microti* and *Borrelia burgdorferi* can be transmitted by the bite of a single coinfected *Ixodes scapularis* tick, an attempt was made to determine the frequency with which whole blood samples that tested positive for *B. microti* infection by polymerase chain reaction (PCR) would also test positive by PCR for *B. burgdorferi* infection. Over a 7-year period from 2013 to 2019, 119 different patients tested positive for *B. microti* infection by PCR on at least one blood sample. Among the 118 patients with a positive *B. microti* PCR blood sample that could also be tested by a qualitative PCR for *B. burgdorferi,* only one patient tested positive (0.85%, 95% CI 0.02 to 4.6%). Routine PCR testing of every *B. microti* PCR-positive blood specimen to detect *B. burgdorferi* coinfection appears to have a low yield, even in a highly endemic geographic area for both of these infections.

## 1. Introduction

The most common cause of babesiosis in humans in the United States is *Babesia microti*, and the most common route of transmission is through the bite of an infected *Ixodes scapularis* tick [1,2]. In certain geographic locations in the United States, including in parts of New York State, the frequency of concomitant *Borrelia burgdorferi* infection in ticks that are infected with the *B. microti* pathogen is over 40% (44% specifically in a recent study [3] that is considered to be representative for the analyses discussed below). Indeed, the rate of *B. burgdorferi* infection in *B. microti* infected nymphal stage *I. scapularis* ticks is often higher than in nymphal stage *I. scapularis* ticks not infected with *B. microti* [3,4]. This is thought to be due to a shared reservoir host and because infection of the reservoir host with *B. burgdorferi* may increase the likelihood of transmission of *B. microti* to a feeding *I. scapularis* larval tick [5].

For symptomatic patients diagnosed with *B. microti* infection, the rate of coinfection with *B. burgdorferi* has been reported to exceed 20% in several studies [6,7], but concerns exist with regard to the accuracy of a diagnosis of *B. burgdorferi* coinfection, unless there is a concomitant objective clinical manifestation of Lyme disease, such as an erythema migrans skin lesion [6]. Indeed, in one case report of an untreated patient with self-resolving untreated babesiosis [6], it was established that false positive antibody testing for IgM antibodies to *B. burgdorferi* may arise, which was transient and not associated with development of IgG antibodies to *B. burgdorferi*. In addition, basing a diagnosis of a Lyme disease coinfection on a positive serologic test alone might simply reflect the patient having had a prior *B. burgdorferi* infection [8].

In this study we evaluated the frequency of a positive polymerase chain reaction (PCR) assay for detection of *B. burgdorferi* on blood samples of patients that tested positive by PCR for *B. microti* on the same blood sample.

## 2. Methods

From 2013 through 2019 as part of a laboratory test development protocol, leftover samples of whole blood specimens submitted to the clinical laboratory for *B. microti* PCR testing were also tested by PCR for evidence of *B. burgdorferi* sensu stricto infection. The blood specimens for PCR testing were collected in a BD lavender-top vacutainer tube with ethylenediaminetetraacetic acid (EDTA) as the anticoagulant. The laboratory is located in the Hudson Valley region of New York State, a geographic area in which both Lyme disease and babesiosis are endemic [9].

The PCR testing for *B. microti* targeted the 18S rRNA gene [10]. In addition to the internal control for each sample, an external negative control and 2 external positive controls (at medium and low target concentrations, respectively) were also included in each PCR run. The *B. microti* PCR assay used will produce a positive result with 95% confidence for a blood specimen with 0.000065% parasitemia. As part of test development, 61 different microorganisms, including 6 *Babesia* species other than *B. microti*, 4 species of *Plasmodium*, and a variety of bacteria, fungi, and viruses that may be found in patient blood samples were tested, and all tested negative. Therefore, the analytical specificity was 100%.

The PCR testing for *B. burgdorferi* was a whole blood qualitative real-time PCR targeting the *B. burgdorferi*-specific 16S rRNA gene, as described elsewhere [11,12]. As per a prior publication [12], the analytical sensitivity of this PCR assay for detection of *B. burgdorferi* strain B31 (ATCC 35210) was one copy per PCR reaction. *B. burgdorferi* strain B31 DNA was used as the positive control for each test run. Since the *B. burgdorferi* PCR testing was intended for laboratory test development only, the test results were not communicated to clinical teams and were not used to influence patient management decisions.

## 3. Results and Discussion

Over the approximate 7-year period from 2013 through 2019, 119 different patients tested positive for *B. microti* infection by PCR on at least one blood sample. Sixty-eight (57.1%) of these patients had two or more positive *B. microti* PCR test results on blood samples collected on different days. One patient of the 119, who only tested positive by PCR for *B. microti* on a single blood sample, was excluded from further analyses, since there was insufficient remaining DNA to also test the same blood sample using the *B. burgdorferi* PCR. Only one of the blood samples, which were PCR positive for *B. microti* from 118 different patients, also tested positive by PCR for coinfection with *B. burgdorferi* (1/118, 0.85%, 95% CI 0.02 to 4.6%). Three days later, another blood sample was obtained from this patient, and again, the patient tested positive by PCR for *B. microti* infection, but this blood sample tested negative by PCR for *B. burgdorferi.* Hypothetically, if antibiotics had been started prior to the repeat blood testing, this might have contributed to the negative result.

In an attempt to understand these findings, a number of issues should be considered in regard to tick transmission of *B. microti* versus *B. burgdorferi*. One question is whether the incubation period from a nymphal *I. scapularis* tick bite until the onset of erythema migrans, the most common clinical manifestation of early Lyme disease [8], differs compared with the onset of clinical symptoms from babesiosis. In the United States, the time to development of an erythema migrans skin lesion following an *I. scapularis* tick bite is in the range of 3 to 30 days [13]. In comparison, the estimated time until development of symptomatic babesiosis following an *I. scapularis* tick bite is in the same range, i.e., 7–28 days [14]. One caveat, however, with regard to assessing the time of onset of babesiosis following a tick bite is that there is no clinical marker at the tick bite site to establish that the tick that was noticed actually transmitted the infection. A second consideration is a comparison of the time from tick attachment to transmission of *B. microti* versus *B. burgdorferi* by ticks that are not coinfected. Of note, a number of similarities exist. Based on animal studies for both infections studied individually, transmission from *I. scapularis* nymphal stage tick bites typically does not occur until at least 36 h following attachment [15,16,17].

Another important question is regarding the frequency of transmission of *B. burgdorferi* to an animal host by coinfected ticks that successfully transmitted *B. microti*. The available data on this question are extremely limited, but based on the only published study to date, the rate of co-transmission was 57% [18]. Using the above-mentioned data, in conjunction with recently published data on the infection rates of 299 nymphal stage ticks from New York State, it can be estimated that out of every 100 ticks that transmit *B. microti*, 44 ticks are coinfected with *B. burgdorferi* [3]. However, for these 44 coinfected ticks, transmission of *B. burgdorferi* might actually only occur for 25 patients (i.e., ~57% based on the limited data from a single animal study [18]). With regard to the rates of blood PCR positivity for *B. burgdorferi* in untreated U.S. patients with erythema migrans, the typical range is 25–50% [8,19,20,21]. Thus, out of the 100 persons hypothetically bitten by a *B. microti* infected tick, as discussed above, only 6 to 13 persons would be expected to test positive by a blood PCR assay for detection of *B. burgdorferi* infection before receiving antibiotic therapy.

An important limitation of the current study is the absence of clinical information in general and, in particular, regarding whether any of the *B. microti* PCR-positive patients had an erythema migrans skin lesion or had already received antibiotics directed to either babesiosis or Lyme disease before the blood sample was obtained. Indeed, if an azithromycin containing anti-babesiosis treatment regimen had been prescribed prior to PCR testing [1], that drug per se would also potentially have had therapeutic efficacy against *B. burgdorferi* [8]. However, a relevant aspect of the data presented here regarding increasing the probability of detecting *B. burgdorferi* coinfection by blood PCR testing is that, for more than 90% of the 118 evaluable cases, the first blood sample that tested positive by PCR for *B. microti* was obtained during the peak months for developing early Lyme disease, i.e., from May through September, 111/118 (94%) cases, including 99/118 (84%) cases who were initially tested from June through August. Another limitation of the study is that genetic sequencing of the *B. burgdorferi* strain found in the single positive blood sample was not performed. In addition, we do not have data on the results of serologic testing for antibodies to *B. burgdorferi*.

With regard to the duration of blood PCR positivity, even successfully treated patients with babesiosis can be expected to test positive for weeks to months [1], but this would seem unlikely to occur with blood PCR testing to detect *B. burgdorferi* infection. Although in our opinion no high-quality published data exist on the persistence of blood PCR positivity for *B. burgdorferi* in early Lyme disease patients who have been treated with appropriate antibiotics, skin PCR testing was found to be positive for *B. burgdorferi* sensu lato for only 1.6% of 61 initially culture-confirmed patients with erythema migrans from Slovenia who underwent a repeat skin biopsy, at or near the original biopsy site, 2-3 months following antibiotic treatment [22]. A potentially important conclusion from the PCR testing for *B. burgdorferi* found in the current study is that persistent blood PCR positivity for *B. burgdorferi* infection is unlikely to actually occur in antibiotic treated patients, even in patients with babesiosis coinfection.

In conclusion, routine testing of all PCR-positive Babesia blood specimens by PCR to detect *B. burgdorferi* coinfection would appear to have a low yield, even in a highly endemic geographic area for both of these infections. Diagnosis of *B. burgdorferi* coinfections in patients with babesiosis, therefore, should not rely on blood PCR testing alone and should be based on other relevant information, such as the presence of a concomitant erythema migrans skin lesion.

## Data Availability

Data not available.
